# Surfactant protein A alters endosomal trafficking of influenza A virus in macrophages

**DOI:** 10.3389/fimmu.2023.919800

**Published:** 2023-03-07

**Authors:** Eric Yau, Linlin Yang, Yan Chen, Todd M. Umstead, Hannah Atkins, Zoe E. Katz, Jonathan W. Yewdell, Chintan K. Gandhi, E. Scott Halstead, Zissis C. Chroneos

**Affiliations:** ^1^ Department of Pediatrics, Division of Neonatal-Perinatal Medicine, Pulmonary Immunology and Physiology Laboratory, Pennsylvania State University College of Medicine, Hershey, PA, United States; ^2^ Department of Comparative Medicine, Pennsylvania State University College of Medicine, PA, Hershey, United States; ^3^ Cellular Biology Section, Laboratory of Viral Diseases, National Institute of Allergy and Infectious Diseases, Bethesda, MD, United States; ^4^ Department of Pediatrics, Division of Pediatric Critical Care Medicine, Pulmonary Immunology and Physiology Laboratory, Pennsylvania State University College of Medicine, Hershey, PA, United States; ^5^ Department of Microbiology and Immunology, Pennsylvania State University College of Medicine, Hershey, PA, United States

**Keywords:** surfactant protein A, macrophages, influenza A virus, lung, collectin

## Abstract

Influenza A virus infection (IAV) often leads to acute lung injury that impairs breathing and can lead to death, with disproportionate mortality in children and the elderly. Surfactant Protein A (SP-A) is a calcium-dependent opsonin that binds a variety of pathogens to help control pulmonary infections by alveolar macrophages. Alveolar macrophages play critical roles in host resistance and susceptibility to IAV infection. The effect of SP-A on IAV infection and antiviral response of macrophages, however, is not understood. Here, we report that SP-A attenuates IAV infection in a dose-dependent manner at the level of endosomal trafficking, resulting in infection delay in a model macrophage cell line. The ability of SP-A to suppress infection was independent of its glycosylation status. Binding of SP-A to hemagglutinin did not rely on the glycosylation status or sugar binding properties of either protein. Incubation of either macrophages or IAV with SP-A slowed endocytic uptake rate of IAV. SP-A interfered with binding to cell membrane and endosomal exit of the viral genome as indicated by experiments using isolated cell membranes, an antibody recognizing a pH-sensitive conformational epitope on hemagglutinin, and microscopy. Lack of SP-A in mice enhanced IFNβ expression, viral clearance and reduced mortality from IAV infection. These findings support the idea that IAV is an opportunistic pathogen that co-opts SP-A to evade host defense by alveolar macrophages. Our study highlights novel aspects of host-pathogen interactions that may lead to better understanding of the local mechanisms that shape activation of antiviral and inflammatory responses to viral infection in the lung.

## 1 Introduction

Influenza viruses are ubiquitous pulmonary pathogens commonly associated with mild upper respiratory infections, but can also result in severe infections of the lower respiratory tract in healthy and vulnerable individuals. Furthermore, there is emerging evidence that influenza coinfection exacerbates SARS-CoV-2 infection and is a suspected etiology of underlying severe COVID19 disease in the pediatric population (1–3). Influenza viruses are members of the *Orthomyxoviridae* family of segmented negative sense single-strand RNA viruses. There are three types, influenza A, B, and C. Influenza A viruses are further classified into 18 subtypes based on the serotype of hemagglutinin (HA) and neuraminidase (NA) on the surface of the viral envelope. Influenza A (H3N2 and H1N1 subtypes) and influenza B seasonal infections as well as periodic pandemic infection caused by influenza A are responsible for substantial mortality, ranging from hundreds of thousands to many millions (4, 5).

Influenza A virus (IAV) infects host cells through attachment of its HA to sialic acid displayed in α2,3 or α2,6 orientation on glycolipid glycoconjugates on the host cell membrane. Attachment is followed by endocytic entry and trafficking to acidified endosomes where HA undergoes conformational change exposing its fusion peptide to merge viral and endosomal membranes to allow for release of viral ribonucleoprotein (vRNP) into the cytosol and then nuclear transport for replication and expression of viral genes and packaging of new vRNPs. HA is synthesized in the cytoplasm by the host ribosomal machinery, followed by transport through the endoplasmic reticulum-Golgi compartments undergoing folding, trimerization, glycosylation, and then localization to the host cell membrane (6, 7). Clustering of HA to cellular membranes along with two other viral envelope proteins, NA and the M2 ion channel, facilitates bud formation and wrapping of the M1 protein capsid that contains prepackaged vRNP segments into the host cell-derived membrane envelope. Virus progeny are then released from the cell-surface *via* cleavage of host sialic acid by influenza NA (8). HA is a trimeric protein consisting of globular and α-helical stalk regions. Newly synthesized HA on virus progeny termed HA0 is cleaved by host proteases into disulfide-linked HA1 and HA2 subunits in the lung’s airway surface fluid, initiating new rounds of infection (9). This cleavage is required for conformational activation of the fusogenic activity of HA in late endosomes. HA1 contains the globular sialic acid receptor binding domain, vestigial esterase, and fusion domains. HA2 contains the transmembrane domain and an ectodomain. The stalk domain comprises the carboxy-terminal and amino-terminal regions of the HA1 and HA2 ectodomains that are subject to structural rearrangements to expose the fusogenic peptide of HA1 at pH 5-6, depending on the influenza strain (9, 10).

Upon reaching the lower respiratory tract, IAV infects alveolar macrophages (AMs) and alveolar epithelial cells, although the two cell-types process and respond to IAV infection differently (11, 12). Other than HA attachment to sialic acid receptors that tether the virus on the respiratory epithelium (13–17), the mechanisms that mediate internalization and recognition of IAV infection by AMs are not well-defined (5, 18). AM dysfunction or depletion impairs survival from IAV infection (19–26). Macrophages, including AMs, support replication and expression of the viral genome but present blocks that limit the production of viral progeny for most IAV strains in mice and humans (27–29), whereas infected epithelial cells package and release high levels of transmissible virus (12). These forms of infection have been termed abortive and productive, respectively. Recent electron microscopy studies of influenza infected human lung, however, showed that monocytes and macrophages in the alveolar space contained influenza A virus only within cytosolic vesicles and found no evidence of nuclear replication of viral genes in these cells *in vivo* (11). In contrast, alveolar type II epithelial (ATII) cells contained viral M1 protein-associated intranuclear dense tubules, indicating that these cells support replication of the viral genome *in vivo* (11).

The interaction of IAV with surfactant proteins A (SP-A) and surfactant protein D (SP-D) may modulate IAV infectivity and biological outcomes in AMs and ATII cells. The latter secrete GM-CSF that is critical for AM differentiation and activation and in host resistance to influenza infection (19, 30–32). SP-A and SP-D are members of the collectin family of proteins that serve in host defense as pathogen opsonins, help maintain immune and surfactant homeostasis, organize structure and function of surfactant proteins and lipids, modulate macrophage activation, and coordinate innate and adaptive immune cells through discrete receptors (19, 33–38). IAV strains with highly glycosylated HA are subject to neutralization by SP-D binding to mannose-rich glycans proximal to hemagglutinin’s sialic acid binding site, neutralizing infection of epithelial cells, and enhancing aggregation, viral uptake and clearance of the virus by myeloid cells (39–42). Antigenic evolution and re-assortment of hemagglutinin gene segments that remove or alter the spatial orientation of mannose-rich glycoconjugate chains deprives SP-D of the ability to neutralize and facilitate clearance of pathogenic strains of influenza (43–46). In addition to evading SP-D binding, paucity of HA glycosylation in pathogenic IAV strains can impair binding to macrophage galactose-type lectin (MGL), DC/L-SIGN, and langerin (47–51). These evasion mechanisms and the absence or inadequate antibody-mediated humoral immunity from prior infection allow new influenza strains to spread in the lower respiratory tract, causing non-resolving inflammatory injury that impairs breathing and gas exchange (52–54).

Although the ability of disease-causing IAV strains to escape neutralization by SP-D is well-established, there is limited information on the role of surfactant protein A (SP-A) in IAV infection (55), despite a large body of evidence indicating that SP-A modulates host defense and inflammation (56). There is renewed interest to illuminate the role of SP-A in IAV infection, as one genetic study showed that certain polymorphisms in the SP-A isoform 2 gene (*SFTPA2*) in humans enhanced susceptibility to H1N1 influenza-induced injury (57). A recent study using the A549 cell line showed that SP-A inhibits infection, whereas a truncated recombinant protein lacking the collagen-like domain enhanced infection (58). In AMs, earlier studies suggest that SP-A acts as an opsonin to enhance uptake of IAV (59). Earlier studies reported that the interaction of SP-A with H3N2 IAV depends on HA binding to terminal sialic acid on its own glycoconjugate neutralizing infection of epithelial cells with both SP-D-sensitive and SP-D–resistant strains of influenza as a γ-inhibitor. This interaction, however, is vulnerable to the action of viral neuraminidase (41, 60, 61). Studies in SP-A-deficient mice suggested increased susceptibility to SP-D-resistant H3N2 infection arising from dysregulation of the immune response (62). Here, we report that SP-A interacts with HA through a protein-protein interaction mechanism and that SP-A alters the endocytic fate of IAV in macrophages in a manner that delays endosomal escape and expression of viral proteins in macrophages.

## 2 Materials and methods

### 2.1 Mice and influenza infection

All animal studies were performed within an American Association for the Accreditation of Laboratory Animal Care-certified barrier facility at Pennsylvania State University College of Medicine. All animal work was approved by the Institutional Animal Care and Use Committee (Protocol No 46031 and 46667). Mice were housed under specific pathogen-free conditions. Mice were provided food and sterile water ad-libitum and maintained on a 12 light/12 dark cycle. SP-A-deficient (*Sftpa^-/-^*) (62–64) and GM-CSF-deficient mice (*Csf2^-/-^*) generated previously (65) on the C57BL/6 genetic background were crossed to generate double-deficient *Sftpa^-/-^, Csf2^-/-^* in house. WT C57BL/6J mice were purchased from Jackson Laboratories (stock number 000664, Bar Harbor, ME) and bred in house. Progeny with heterozygous or homozygous deletion in one or both alleles were genotyped either in-house by PCR or commercially by quantitative real time PCR using DNA template of tail biopsies collected from 3-week-old pups (Transnetyx, Inc, Cordova, TN). Primer sequences utilized for in-house PCR genotyping for *Sftpa^-/-^* mice were 5’-GCT ACT TCC ATT TGT CAC GTC C-3’ (*Sftpa* mutant reverse primer) and 5’-ACA GAA GTT TGT GCC GGA AG-3’ (*Sftpa* forward primer common to mutant and WT allele) and 5’-ATG GTC ACC CAG AAA ACA GG-3’ (*Sftpa* mutant reverse primer). PCR with this primer set yields 320 and 167 base pair amplicons for mutant and WT alleles. The PCR primer sequences for *Csf2^-/-^* mouse genotyping was 5’- AGGTCT TCA GGG ATT GAT GG-3’ (CSF2 common), 5’- CTCAGC TAC CAC AGC CAT GT-3’ CSF2 (WT reverse primer), and 5’- CTCCAG ACT GCC TTG GGA AAA-3’ (CSF2 mutant reverse primer). This primer set yields 300 and 317 base pair amplicons for the mutant and WT alleles, respectively.

Deletion of SP-A was confirmed by Western blot analysis of bronchoalveolar lavage fluid (**Supplemental Figure S1A**). Deletion of GM-CSF was confirmed by turbidity (**Supplemental Figure S1B**) and protein concentration in bronchoalveolar lavage (**Figure S1C**) for the presence of alveolar proteinosis (65, 66). Studies utilized both male and female mice at 8-12 weeks of age (30). Mice were infected with 1000 fluorescent focus units (ffc) of the mouse adapted H1N1 strain A/Puerto Rico/8/13 (PR8) in 40 μl of PBS *via* the intranasal route under anesthesia induced *via* intraperitoneal injection of a ketamine (100 mg/Kg)/Xylazine (10 mg/Kg) mixture. Infected mice were weighed daily and monitored for clinical symptoms as detailed previously (30). Viral titer and expression of *Ifnb* mRNA was determined in whole lung by quantitative real time reverse transcriptase PCR as described in detail previously (67). Histopathology of hematoxylin and eosin tissue sections from infected lungs was evaluated by a board-certified veterinary pathologist blinded to genotype. Lungs were processed for histopathology evaluation and scoring as described in detail previously (67).

### 2.2 Cell culture

The RAW264.7 peritoneal macrophage cell line (ATCC Cat No CRL 2278) (68–70) was maintained in DMEM culture media (DMEM with 4.5 g/L glucose, L-glutamine, and sodium pyruvate supplemented with 10% heat-inactivated fetal bovine serum (FBS) and 1% penicillin/streptomycin) at 37°C and 5% CO_2_ in 10-cm dishes.

### 2.3 Virus preparation and quantitation

The mouse adapted IAV H1N1 strain A/Puerto Rico/8/34 (PR8) and human H3N2 strain A/Philippines/82 (Phil82) and a PR8 recombinant strain with a carboxy-terminal non-structural protein 1 (NS1) fusion with green fluorescent protein (PR8-GFP) (71) were propagated in the allantoic fluid of embryonated chicken eggs (31). Briefly, 10^5^ fluorescent focus units (FFC) of PR8 IAV in PBS with 1% Penicillin/Streptomycin/Fungizone was injected into the amniotic sac of 10 day old embryonated chicken eggs. Infected eggs were incubated at 37°C for 56 hours. Eggs were then removed and placed at 4°C for 12 hours. The allantoic fluid was collected and spun down at 131,000g for 40 minutes at 4°C. The virus pellet was reconstituted in PBS and layered over a 30%/35%/50%/60% sucrose gradient and spun at 168,000xg for 1 hour and 15 minutes and the virus containing layer between 50% and 35% was collected and dialyzed against PBS at 4°C overnight. Viral titer was determined by fluorescent focus count (ffc) assays using Madin-Darby Canine Kidney (MDCK) epithelial cells (ATCC Cat No CRL-2936). MDCK cells were plated in 96-well tissue culture dishes at a density of 3x10^4^ per well and overlaid with serial dilutions of purified and dialyzed virus. Cells were then incubated at 37°C with virus for 2 hours, at which point virus-containing media were replaced with virus-free media and cells were cultured for another 6 hours. Cells were then fixed with acetone and stained with an IAV NP antibody (Sigma Aldrich Cat No MAB8251, 1:100 in PBS) for 30 minutes at 4°C and subsequently labeled with Rhodamine conjugated anti-mouse IgG (Jackson ImmunoResearch, Cat No 115-026-062, 1:100 in PBS). Fluorescently labeled nuclei were counted using a Nikon Eclipse TE2000-U at 20x magnification. Viral load in lung was determined by qRT-PCR exactly as detailed previously (67).

### 2.4 SP-A purification

Human SP-A was isolated *via* butanol extraction as described by Haagsman et al. (72). Briefly, human alveolar proteinosis fluid from bronchoalveolar lavage (AP BAL) was thawed and added dropwise to a volume of stirring butanol at a ratio of 15:1 mL of butanol to AP BAL. The mixture was stirred for 30 minutes after the entire volume of AP BAL was added; additional butanol was added dropwise until only 1 phase is present. The mixture was then centrifuged at 5000xg for 30 minutes at 15°C with a Beckman J-21C centrifuge. Supernatant was carefully discarded, and process was repeated until entire volume has been pelleted. The pellet was then completely dried with flux of N_2_ gas. Dried pellets were stored at -20°C until further processing. The pellets were homogenized in 24 mL of 20 mM OGP (n-Octyl B-D glucopyranoside, Sigma Cat No O-8001), 10mM HEPES, and 150 mM NaCl solution at pH 7.4. Homogenized pellet was transferred to polypropylene centrifuge tubes and allowed to sit for 30 minutes. After 30 minutes, the homogenized solution was centrifuged at 210,000xg using a Beckman L5-65 centrifuge for 30 minutes at 15°C. After centrifugation, supernatant was discarded and the homogenization and centrifugation were repeated. After the second centrifugation, the pellet was resuspended in 10 mL of 10 mM HEPES, transferred to a 7000 MWCO dialysis membrane (ThermoFisher Cat No 68700), and dialyzed against 10 mM HEPES overnight at 4°C. After dialysis, dialyzed material was centrifuged at 200,000xg for 30 minutes at 4°C and the supernatant was collected. Supernatant was assessed for SP-A concentration *via* BCA assay (ThermoFisher, Cat No 23235), and purity was determined *via* gel electrophoresis and analysis by Coomassie stain and SP-A specific blotting. Endotoxin contamination was assessed *via* LAL chromogenic assessment kit (ThermoFisher Cat No A39552).

### 2.5 SP-A treatment

Cells were incubated with SP-A at a concentration of 50 μg/mL unless otherwise noted in Figure legends. SP-A was diluted using DMEM with 10% FBS before 250 μL of the mixture was added to the cells; SP-A in DMEM with 10% FBS was incubated with the cells for 5 hours before IAV infection unless otherwise indicated. Preformed SP-A-IAV complexes were obtained by incubation of SP-A and IAV for 2-hours at room temperature in PBS prior to addition to cells. In the case of IAV infection, SP-A was added at a concentration of 50 μg/mL to the PBS/DMEM media containing IAV and allowed to incubate with the cells at 37°C.

### 2.6 De-glycosylation of SP-A and HA

De-glycosylation of SP-A and HA (BEI Resources, Cat No NR19240) was performed according to manufacturer’s protocol for N-glycosidase F (Sigma Aldrich, Cat No 11365177001). Briefly, 1 µL of N-glycosidase F per 8 µg of SP-A or HA in 10 mM HEPES was incubated at 37°C for 24 hours. After incubation, samples were concentrated, washed and buffer exchanged with 20 mM MES buffer at pH 6.5 *via* micron 10k centrifugal filter (EMD Millipore, Cat No MRCPRT010) at 14,000 RCF. Samples were resuspended in 500 µL of 20 mM MES and protein concentration was determined by BCA assay (ThermoFisher, Cat No 23235).

### 2.7 Solid phase plate assay

Glycosylated or de-glycosylated HA or BSA as a control was coated on a HB 96 well microtiter plate overnight at 4°C using 0.1M carbonate/bicarbonate buffer. Wells were washed with PBS and blocked with 5mM HEPES at pH 7.4 with 150 mM NaCl and 5 mg/mL of BSA. Glycosylated or de-glycosylated SP-A were added at concentrations of 10, 25, 50, 75 and 100 μg/mL in blocking buffer with 2 mM Ca^+2^ and incubated for 1 hour at 37°C. After washing, plates were incubated with 1:5000 dilutions of rabbit anti-SP-A polyclonal antibodies followed by washing and incubation with 1:10,000 dilution of an HRP-conjugated anti-rabbit antibody (Bio-Rad Cat No 170-6515). Bound protein was visualized calorimetrically at 450 nm using tetraethyl benzidine (R&D Systems, Cat No DY999) as the HRP substrate and color development stopped using 2M H_2_SO_4_. Plates coated with albumin were used as controls.

### 2.8 Surface plasmon resonance

Surface plasmon resonance (SPR) experiments were performed using a 2-channel OpenSPR instrument (Nicoya Lifesciences, Kitchener, ON) with SP-A and histidine tagged PR8 HA as analyte and ligand, respectively. We determined the binding kinetics of the SP-A with the H1 Hemagglutinin (HA) Recombinant A/Puerto Rico/8/1934 (H1N1) HA (PR8) expressed in insect cells from a baculovirus vector with a carboxy-terminal hexa-histidine tag was obtained from BEI resources (cat no NR-19240) (73). SPR assays were performed at 20°C in PBS 0.01% Tween-20 (PBST). Histidine tagged HA was immobilized on a nitrilotriacetic acid (NTA) sensor chip for 10 min at a flow rate of 10 μL/min at a concentration of 50 µg/ml following EDTA conditioning. Histidine-tagged streptavidin (Abcam, Cat: ab78833) was immobilized in the reference channel as a control ligand. SP-A was diluted in PBST with and without 0.5 mM calcium and injected for 5 min at a flow rate of 20 μL/min in a concentration range from 10 to 80 µg/ml, with 10 min dissociation time. The sensor chip was regenerated using 200 mM imidazole and 10 mM glycine–HCl (pH 1.5) and reused no more than two times. The resonance intensity of channel 2 was corrected by subtracting that of channel 1, and the kinetic curves were fitted to the one-to-one model using TraceDrawer analysis software (Ridgeview Instruments, Uppsala, Sweden) to derive kinetic, k_on_, k_off_, and binding, K_D_, parameters of SP-A-IAV interaction.

#### 2.8.1 SP-A and IAV binding to isolated cell membranes

Cell membranes were isolated from RAW264.7 cells by osmotic lysis, homogenization, and high-speed centrifugation as described in detail previously (38). Cell membrane protein was quantitated using BCA assays and stored at -80^0^C. For binding assays, Immulon 2 flat-bottom plates were coated overnight with 12.5 μg/mL cell membrane diluted in PBSCM in plates pre-incubated with Invitrogen ELISA coating buffer (Cat No: 00-0044-59) at 4^0^C in 100 μL volume per well. Plates were washed twice with 300 μL/well of 1 x calcium and magnesium replete PBS (PBSCM) (Corning Cat No 46-013-CM) and then blocked for 1-hour in PBS/1% BSA. Blocked plates were washed in 300 μL/well PBSCM and incubated with 50 μg/mL SP-A, preformed SP-A (50 μg/mL)-IAV (5 x 10^6^ ffc IAV/mL), 5 x 10^6^ ffc IAV/mL, PBSCM (with calcium and magnesium) or PBS (without calcium and magnesium) for 2-hours at room temperature. Plates were then washed twice and re-incubated with IAV, SP-A+IAV, SP-A as indicated on Figure and legend 4F in results. After washing, bound virus was detected by incubation with a 1:2000 dilution of polyclonal rabbit anti-HA antibody (Invitrogen Cat No MA5-29931) in 100 μL PBSCM/0.1% BSA for 1-hour at room temperature, washed, and incubated with 1:2500 dilution of HRP-conjugated anti-rabbit IgG (Bio-Rad Cat No 1706515) for 1-hour in PBSCM/0.1% at room temperature. Bound antibody was visualized by addition of 100 μL of R&D Systems color reagent A and B 1:1 mix (Cat No 895000 Reagent A and 895001 Reagent B), reaction stopped with 50 μL R&D Systems stop solution (Cat No 895926), and quantitated spectrophotometrically using SpectraMax M3 spectrophotometer.

### 2.9 Ligand blotting assays

Ligand blotting was performed using biotinylated SP-A (bSP-A) on Western blots containing electrophoretically transferred protein as described in detail previously (38). Briefly, SP-A was biotinylated in MES buffer, pH 6.0 and used for blotting at 1.3 μg/mL in TBS-T buffer (25 mM Tris, pH 7.6, 0.146 M NaCl, 0.1% Tween-20 and 5% BSA). Bound bSP-A was visualized by sequential incubation of washed blots with horseradish peroxidase (HRP)-conjugated Streptavidin and 3,3’-diaminobenzidine as the HRP substrate. Blots were developed using enhanced chemiluminescence. For blotting, purified baculovirus expressed HA or NA were obtained from BEI Resources with cat no NR-34587 (A/Toulon/1173/2011 (H1N1)pdm09 HA), NR-19240 (A/Puerto Rico/8/1934 (H1N1) HA), NR-19241 (A/New York/55/2004 (H3N2) HA), NR-2633 (A/Netherlands/219/2003 (H7N7), NR-41792 (A/Hong Kong/33982/2009 (H9N2) HA), NR-43739 (A/duck/Hunan/795/2002 (H5N1) HA), and NR-42002 (A/Puerto Rico/8/1934 (H1N1) NA). For Western blotting, 0.1 μg of recombinant protein in reducing NuPAGE LDS sample buffer (ThermoFisher, Cat No NP008) were loaded onto a 10-well 4-12% NuPAGE Bis-Tris SDS-PAGE gel (ThermoFisher, Cat No NP0322BOX), electrophoretically separated and blotted onto Nitrocellulose. The PageRuler Plus Prestain Protein Ladder (ThermoFisher, Cat No 2669) was used as molecular weight standard.

### 2.10 Influenza infection and assessment of infection

Raw264.7 cells were seeded at a density of 2 or 3x10^5^ per 24-well for 12-24 hours prior to infection. Cells were then washed with PBS twice and PR8 virus was added at desired multiplicity of infection (MOI) in infection media (1:1 ratio DMEM w/o serum to PBS) as determined by cell seeding density unless otherwise indicated. Cells were incubated with virus-containing infection media at 37°C for two hours. Infection medium was then removed and replaced with DMEM/10% FBS culture media. Infection was allowed to progress at 37°C until the desired incubation time was reached. Incubation time was determined based upon time at which virus was initially added; if incubation was under 2 hours, virus-containing media was not replaced, and cells were washed and harvested at the indicated time. IAV infection was assessed *via* viral protein production by Western blotting, flow cytometry, or confocal immunofluorescence microscopy as described below.

### 2.11 Immunofluorescence

Immunofluorescence of IAV-infected cells was performed on PR8 infected cells at MOI10 after 4 hours of infection. Cells were washed with PBS twice and then blocked with 10% donkey serum and 3% BSA in PBS for 20 minutes at 4°C. Blocked cells were then washed with PBS twice, stained with AlexaFluor488 conjugated Cholera Toxin B subunit (Molecular Probes Cat No C34775, 1:400) in 1% donkey serum and 0.3% BSA in PBS for 30 minutes at 4°C. Cells were then washed, fixed using 3.7% formaldehyde for 25 minutes, and then permeabilized in 0.3% Triton X-100 in PBS for 15 minutes. Fixed and permeabilized cells were blocked with 10% donkey serum and 3% BSA in 0.2% Tween 20 in PBS for 20 minutes at room temperature. Blocked cells were probed using NP (EMD Millipore Cat No MAB8257, 1:400) in 1% donkey serum and 0.3% BSA in 0.2% Tween 20 in PBS overnight at 4°C, and labeled AlexaFluor594 conjugated anti-rabbit IgG (Jackson ImmunoResearch Cat No 711-585-152, 1:500) in 1% donkey serum and 0.3% BSA in 0.2% Tween 20 in PBS for 2 hours at room temperature. Slides were mounted using Prolong Diamond Antifade mountant with DAPI (ThermoFisher Cat No P36962). Images were acquired using Nikon Eclipse T*i* confocal microscope and processed using Nikon Elements 4.30.01 software.

### 2.12 Fluorescent labeling of IAV

PR8 virus was labeled according to manufacturer’s protocol with NHS Ester AlexaFluor-488 (ThermoFisher, Cat No A20000) (74).

### 2.13 Endocytic assay

For experiments utilizing pHrodo-labeled cargo, cells were washed and incubated with 125 μL cargo-containing 1:1 PBS/DMEM media for 15 minutes at room temperature. pHrodo Green Dextran MW10000 (ThermoFisher, Cat No P35368) and pHrodo Red Transferrin (ThermoFisher, Cat No P35376) were used at 30 μg/mL. After incubation, media were removed, cells washed, and 200 μL of 1:1 DMEM/PBS was added. Cells were then incubated at 37°C for the specified time (10, 20, 30, or 45 minutes). Cells were washed and 300 μL of PBS with 2% FBS and 0.02% sodium azide was added. Cells were removed *via* cell scraper and fluorescence data was collected using an LSRII or Fortessa flow cytometer and analyzed using FlowJo 9.8.8. For experiments utilizing FITC-Dextran (ThermoFisher, Cat#D1820), AlexaFluor488-Transferrin (ThermoFisher, Cat No T13342) or AlexaFluor488 labeled IAV PR8, cells were washed and incubated with 125 μL of cargo-containing 1:1 PBS/DMEM media. Cells were placed at 37°C for the specified time (5, 10, 15, 30 or 45 minutes) without preincubation at room temperature. Cells were harvested at time points using a cell scraper, and placed on ice as described above. Flow cytometry data were collected using an LSRII or Fortessa flow cytometer and analyzed using FlowJo 9.8.8.

### 2.14 Western blot analysis

Cells were harvested using 0.25% Trypsin-EDTA (Corning Cat No 25-053-Cl and pelleted by centrifugation at 15,000xg for 5 minutes at 4°C (Eppendorf 5430R). Cell pellets were placed at -20°C overnight with 60 μL SDS Lysis buffer (1% SDS, 50mM Tris-HCL pH 8.1, 10mM EDTA pH 8.0) with 1x Protease/Phosphatase Inhibitor Cocktail (Cell Signaling Cat No 5872S). Samples were thawed and sonicated at 50% amplitude for 10 seconds in 2 second ON/OFF interval using a Branson SFX150 Cup Horn instrument. Sonicated samples were centrifuged at 15,000xg for 5 minutes at 22°C. Supernatant was collected; 4x LDS sample buffer (Invitrogen Cat No NP007) was added to a final concentration of 1x. For gel electrophoresis, samples were diluted to 1X LDS-PAGE sample buffer, boiled at 95°C for 2 minutes, and centrifuged for 2 minutes at 21,000xg. Equal volumes were loaded in 4-12% Bis-Tris NuPAGE reducing gels (Invitrogen, Cat No NP0322BOX, NP0349BOX) in MOPS running buffer (cat No NP0001). Gels were transferred to immobilon-FL PVDF membranes using the GenScript eBlot L1 System. Blots were blocked in 5% Bovine Serum Albumin (BSA) in 0.1% Tween 20 in Tris-buffered saline (0.1% TBST). Blots were probed at 4°C overnight with NS1 (Invitrogen Cat No PA532243, 1:1000), NP (Bio-Rad Cat No MCA400, 1:1000), HA (InVitrogen Cat No MA5-29931, 1:2000), or in-house rabbit anti-human SP-A antibody (1:1000), or mouse anti-actin (Sigma Cta No A1978-200 UL, 1:2,000) in 0.1% TBST and subsequently incubated with IRDye 680RD anti-Mouse IgG (LI-COR Cat No 926-68070, 1:15000) and IRDye 800 CW anti-Rabbit IgG (LI-COR Cat No926-32211, 1:10000) in 0.1% TBST for 2 hours at room temperature. Blots were imaged using LI-COR Odyssey CLx and Image Studio 4.0. Band densitometry was acquired and images were adjusted using Image Studio Lite 5.2.5.

### 2.15 Flow cytometry

Cells were detached using 0.25% Trypsin-EDTA (Corning Cat#25-050-Cl) and washed in PBS containing 2% fetal bovine serum (FBS) *via* centrifugation and discarding of supernatant. Cells were fixed using 100 μL IC Fixation Buffer (eBioscience Cat No 00-8222-49) for 15 minutes. Cells were washed and then blocked with mouse Fc block (BD Biosciences Cat#553142) in PBS at a concentration of 12.5 μg/mL and 2% FBS for 10 minutes at room temperature. After blocking, cells were washed once with 1x permeabilization buffer (eBioscience Cat No 00-8333-56) and then incubated with 1x permeabilization buffer for 5 minutes before pelleting the cells and discarding the supernatant. The cell pellet was resuspended and stained with FITC-NP (EMD Millipore Cat No MAB8257F, 1:40) in 1x permeabilization buffer for 30 minutes at 4°C to assess for IAV nucleoprotein (NP) production or a monoclonal antibody (clone Y8-1C1) recognizing a pH sensitive HA epitope on PR8 in acidified endosomes and newly synthesized protein in the endoplasmic reticulum (75, 76). Cells were washed once more with 1x permeabilization buffer and resuspended in HBSS with 2% FBS and 0.02% sodium azide until assessment *via* BD LSRII or BD FACS Symphony flow cytometer instruments. A minimum of 30,000 events were collected and data were analyzed *via* FlowJo 9.9.5 or 9.8.8 by gating to include singlets and exclude debris in the analysis and then generate single parameter histograms by gating on NP+ events to obtain the percentage of NP+ cells (**Supplemental Figures S2A and B**).

### 2.16 Statistical analysis

Graphs, survival curves, and statistical comparison of data were performed using GraphPad Prism 9.5.0 software (San Diego, CA). 2-way ANOVA with paired samples and unpaired comparisons *via t*-test corrected by the Holm-Sidak method were used to assess statistical differences. p-values<0.05 were considered significant.

## 3 Results

### 3.1 SP-A attenuates IAV infection in macrophages

**Figure 1:**
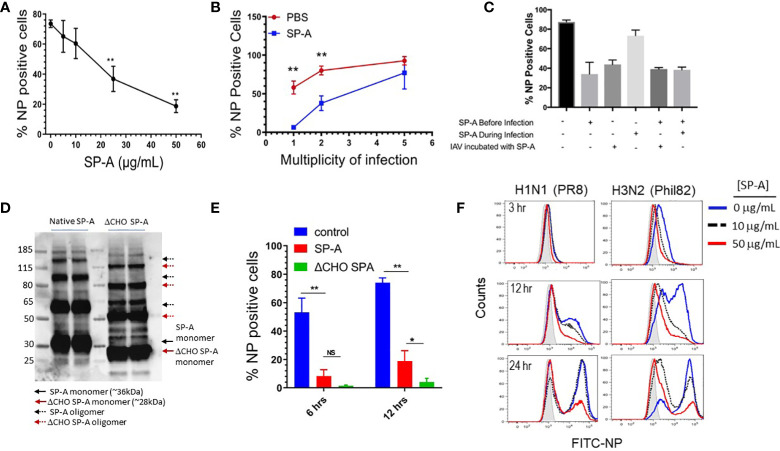
SP-A inhibits IAV infection independent of its glycosylation. RAW264.7 cells were cultured overnight at a density of 2 x 10^5^ cells/well in 24-well plates and infected with either IAV PR8 **(A-F)** or IAV Phil82 **(F)**. Cells were incubated with indicated concentration of virus in 1:1 (v/v) PBS/DMEM and then allowed to proceed in DMEM/10% FBS. At the endpoint of the following experiments cells were harvested, stained with NP antibodies, and analyzed by flow cytometry. **(A)** SP-A inhibits IAV infection in a concentration-dependent manner. RAW264.7 were pretreated with increasing concentration of 5, 10, 25 and 50 μg/mL SP-A for 5 hours and then infected with IAV PR8 at MOI=2 in the presence of respective concentration of SP-A. The percentage of infected cells was determined by flow cytometry 10-hous after infection. N=4, t-test, **p < 0.05. **(B)** IAV overcomes SP-A inhibition at high MOI. RAW264.7 cells were incubated in the presence or absence of 50 μg/mL of SP-A for 5 hours, then infected with IAV PR8 at MOI 1, 2, and 5 in 1:1 PBS/DMEM in the presence or absence of 50 μg/mL SP-A and infection was assessed by flow cytometry. N=6, t-test, **p < 0.05 **(C)** Either pretreatment of macrophages or IAV with SP-A inhibits infection. RAW264.7 cells were incubated with 50 μg/mL SP-A in indicated combinations before or during infection. N=2 independent experiments per condition. **(D)** De-glycosylation of SP-A was accomplished using EndoF and confirmed by visualization of molecular weight reduction of monomeric and oligomeric forms of SP-A on Western blots using a polyclonal SP-A antibody. **(E)** The inhibitory effect of SP-A does depend on its glycoconjugate moiety. De-glycosylation enhances the inhibitory effect SP-A on IAV infection. RAW264.7 cells were treated with either native of de-glycosylated (ΔCHO) SP-A and infected with IAV at MOI=2 and infection assessed at 6 and 12 hrs. after infection. N=4, t-test, *p < 0.05; **p < 0.01, NS, not significant. **(F)** SP-A delays temporal infection with both H1N1 and H3N2 strains of IAV. RAW264.7 were infected with either IAV H1N1 strain PR8 or H3N2 strain Phil82 following a 5-hour treatment with no or either 10 or 50 μg/mL SP-A. Infection was then assessed by flow cytometry at 3, 12, or 24 hours after infection. Representative histograms of n=2 independent experiments per treatment are shown.

The RAW264.7 macrophage cell line has been used extensively to define the life cycle of a wide range of influenza strains (29) and as an AM surrogate cell line to investigate the role of SP-A in endocytic processes (37, 61, 77). Here, RAW264.7 macrophages were treated with various concentrations of SP-A for 5 hours, then subsequently infected with IAV at MOI of 2 in the presence of SP-A. The virus-containing media was removed and the infection was allowed to proceed at 37°C for a total of 12-hours, then harvested and analyzed for production of IAV nucleoprotein (NP) as a measure of infection. **Figure 1A** shows that SP-A reduced infection in a concentration-dependent manner and up to 75% at the highest concentration tested. Conversely, keeping the SP-A concentration constant at 50 μg/mL while increasing the MOI showed that the virus could overcome the inhibitory activity of SP-A at high MOI (**Figure 1B**). We then assessed whether timing of SP-A incubation impacts IAV infection (**Figure 1C**). Incubation of macrophages with SP-A prior to virus addition, but not co-addition at the same time with virus, suppressed infection by 35%. Incubation with SP-A before and throughout IAV infection suppressed infection (**Figure 1C**). Pre-incubation of the virus with SP-A, however, prior to adding the virus to the macrophages, irrespective of whether the macrophages were pre-treated with SP-A, also suppressed infection (**Figure 1C**). Furthermore, the inhibitory activity of SP-A was not dependent on its decoy glycoconjugate (**Figures 1D, E**). De-glycosylated SP-A (**Figure 1D**) was more potent at inhibiting infection (**Figure 1E**). **Figure 1F** shows that SP-A inhibited infection with both H1N1 and H3N2 strains of influenza as shown by flow cytometry staining using the nuclear protein (NP) antibody. The inhibitory effect was sustained for up to 24-hours at 50 μg/mL. These results indicate that SP-A can inhibit binding of the virus to macrophages by binding either the virus or macrophages prior to infection.

### 3.2 SP-A binds IAV hemagglutinin in a glycosylation-independent manner

**Figure 2:**
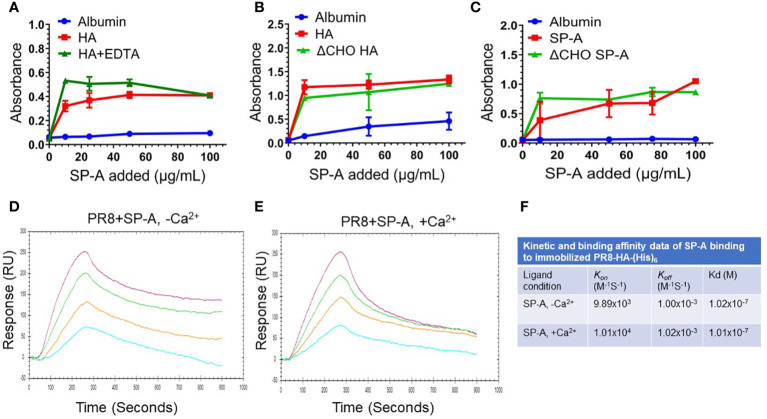
SP-A binds HA in a calcium and glycosylation independent manner. SP-A binding to HA was carried out in static solid phase assays **(A-C)** and under flow using surface plasmon resonance (SPR) **(D-F)**. For static assays, recombinant PR8 HA (1 μg/well) was adsorbed onto 96-well Immobilon-2 plates in 0.05 M bicarbonate buffer (pH 9.6) overnight at 4°C. Coated plates were incubated with albumin control or SP-A at room temperature for 1 hour. Plates were washed in binding buffer and incubated with a polyclonal SP-A antibody. Bound SP-A was visualized colorimetrically using HRP-conjugated anti-rabbit antibody and tetramethylbenzidine. Albumin was used as non-specific control. **(A)** SP-A binding to HA does not depend on the presence of calcium. HA and albumin control coated plates were washed and incubated with increasing concentration of 10, 25, 50, and 100 μg/mL of SP-A in the presence or absence of 5 mM EDTA. N=4 of 2 independent experiments. **(B)** De-glycosylation (ΔCHO) of HA does not prevent SP-A binding. Plates coated with ΔCHO recombinant HA, or albumin were washed, and then incubated with increasing concentration of 10, 50, and 100 μg/mL of SP-A. N=2. **(C)** De-glycosylation (ΔCHO) of SP-A does not prevent binding to HA. Recombinant HA or albumin control coated plates were incubated with increasing concentration of 10, 50, 75, and 100 μg/mL of SP-A or ΔCHO SP-A. N=4 of 2 independent experiments. **(D-F)** Calcium does not impact kinetic and binding parameters of SP-A binding to HA. For SPR assays, recombinant HA with a carboxy-terminal hexa-histidine tag was immobilized onto Nicoya nitrilotriacetic acid (NTA) sensor chips. Increasing concentration of 10, 20, 40, or 80 μg/mL of SP-A analyte in the presence **(D)** or absence **(E)** of 0.5 mM CaCl_2_ was injected at 20 μL/min for 300 sec to obtain on-rates and switched to buffer without SP-A for an additional 600 sec to obtain off-rates. Binding sensograms were acquired using a 2-channel Nicoya OpenSPR instrument. Curves were fitted according to 1:1 interaction model to obtain kinetic and binding parameters **(F)**. Data shown are representative of N=3 independent experiments.

The findings shown on **Figure 1C** above show that pre-incubation of IAV with SP-A before addition to the cells suppresses infection, indicating that SP-A interacts directly with the virus in a manner that is inhibitory to subsequent infection. We used ligand blot and solid phase assays to evaluate whether SP-A binds viral membrane proteins HA and neuraminidase (NA). Ligand blotting using biotinylated SP-A and blotted recombinant proteins showed that SP-A interacts with HA from different IAV strains (**Supplemental Figure S3**, lanes 1-5, 7) but not NA (**Supplemental Figure S3**, lane 6). Solid phase assays were then used to characterize the interaction of SP-A with PR8 HA (**Figure 2**). PR8 H1N1 lacks carbohydrate chains on the globular sialic acid binding domain of the HA1 subunit, but retains carbohydrate chains in the stem domain (42, 78). The presence of EDTA (**Figure 2A**) or de-glycosylation of HA (**Figure 2B**) did not block SP-A binding. Furthermore, both native and de-glycosylated SP-A bound HA at concentration greater than 10 μg/mL (**Figure 2C**), suggesting that its carbohydrate chain contributes to optimal binding at low concentration of SP-A (59). SP-A did not bind albumin coated plates (**Figures 2A-C**). These results indicate that SP-A binds HA through a lectin- and glycoconjugate independent mechanism. We then used surface plasmon resonance (SPR) to obtain the SP-A-HA binding parameters (**Figures 2D-F**). Recombinant HA with a carboxy-terminal hexa-histidine tag was immobilized onto NTA sensor chips and SPA injected at increasing concentration in the presence or absence of calcium. **Figures 2D, E** show that SP-A binding increased in a concentration-dependent manner and that binding is not calcium-dependent, consistent with the static solid phase assay results above. SP-A exhibits similar on and off rates and binding affinity in the presence and absence of calcium (**Figure 2F**).

### 3.3 SP-A delays IAV infection at the endosomal level

**Figure 3:**
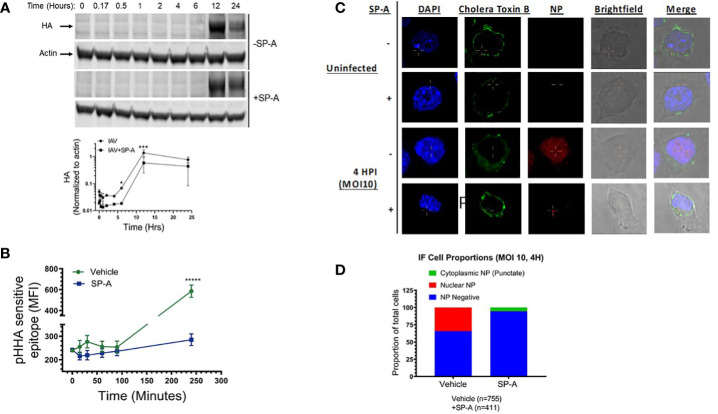
SP-A delays IAV infection at endosomal level. **(A)** SP-A delays synthesis of HA. RAW264.7 cells were treated with SP-A for five hours and then infected with IAV at MOI=15. Cells were harvested 1, 2, 4, 6, 12, and 24-hours after infection to obtain cell extracts for Western blot analysis using a polyclonal rabbit anti-HA antibody and a fluorescent IRDye800CW goat anti-rabbit secondary antibody. Blots were probed with an anti-mouse actin antibody and IRDye680RD goat anti-mouse antibody as loading control. Blots were quantitated by fluorescence densitometry using a LICOR instrument to obtain band intensity graphs and densitometry data for HA band intensity were normalized to actin. N=3, *p < 0.05 and ***p < 0.001 assessed by 2-way ANOVA. **(B)** To monitor trafficking to acidified endosomes, RAW264.7 cells were treated with SP-A for 5 hours and infected with IAV MOI=15 for 15, 35, 60, 90 and 240 minutes. Cells were harvested, fixed, permeabilized, and stained with an antibody recognizing a pH-dependent epitope that is exposed upon conformational transition of HA to the fusion competent form (pHHA). SP-A suppressed detection of the low pH HA conformation over the first 30 min of infection and delayed synthesis of new HA. N=4, *****p < 0.00005 assessed by tailed t-test. **(C, D)** SP-A treatment results in reduced infection and retention of NP in a punctate perinuclear compartment. Immunofluorescence microscopy was used to visualize the effect of SP-A on localization of viral NP. Cells were treated with 50 μg/mL SP-A for 5 hours and then infected with IAV for 4 hours. Cells were then fixed and stained with FITC-Cholera Toxin B subunit to stain the cell membrane, antibodies to NP to visualize IAV, and DAPI to stain the nucleus **(C)**. Stained cells were counted across 10 microscopic fields at 63x magnification using a Nikon Eclipse confocal microscope to obtain the distribution of NP between nuclear and cytosolic compartments **(D)**.

The findings shown on **Figures 1A-F** above, show that pre-incubation of macrophages with SP-A also attenuates infection, indicating that SP-A influences the fate of the un-opsonized virus in macrophages. To further understand the role of SP-A on uptake of the virus, we monitored expression of HA by Western blot analysis overtime after pre-incubation of the cells with SP-A (**Figure 3A**). SP-A delayed expression of HA by 2-hours following infection with high MOI of 15. At this MOI, IAV can overcome the inhibitory effect of SP-A (**Figure 1C**). The densitometry data on **Figure 3A** show that HA expression rose sharply after 4-hours in the absence of SP-A compared to 6-hours in the presence of SP-A (**Figure 3A**). Differences were statistically significant at 6- and 12-hours after infection. HA levels appear depressed prior to 6-hours in the presence of SP-A compared to the absence of SP-A (**Figure 3A** densitometry), suggesting initial virus clearance or reduced uptake, although there was not sufficient signal strength at early stage of infection to determine statistical differences. A similar lag was detected for NS1 1-2-hours after infection, although NP kinetics were similar (**Supplemental S4**). In this regard, NS1 translated at early stage of infection is required for expression of HA but not NP (79, 80). The delayed kinetics of HA and NS1 expression even at high MOI suggested an endosomal trafficking or earlier block since NS1 is not incorporated into viral progeny, and thus not part of internalized virus. To assess whether SP-A alters infection at the endosomal level, we used an antibody against a pH-dependent epitope on IAV HA (referred here as pHHA) to monitor the pH sensitive conformational transition of HA in acidified late endosomes (75, 76). To have a greater signal, untreated cells were infected with IAV at MOI 15, which showed an initial increase in signal of the pHHA antibody through the first 30 minutes of infection, consistent with IAV trafficking to acidified endosomes and the subsequent unfolding of the HA trimer to the fusion competent form (**Figure 3B**). The pH sensitive epitope peaked 30 minutes after infection, followed by decline over the next 60 minutes in control cells. This was then followed by an increase in the pHHA antibody-bound epitope over the next 4 hours, associated with new HA synthesis (**Figure 3B**). In contrast, SP-A impaired detection of the pH-sensitive epitope signal over the first 30 min of infection. Subsequently, SP-A treated cells showed a slow, continuous increase in signal intensity between 15 minutes to 4 hours post infection (**Figure 3B**). To evaluate endosomal uptake, cells treated with either vehicle or SP-A were stained for viral NP and AlexaFluor 488 conjugated cholera Toxin B subunit to visualize internalized IAV and the cell membrane 4 hours after infection, respectively. Confocal imaging of infected cells revealed that SP-A treated cells showed NP-positive puncta within the boundary of cholera toxin B staining, indicative of internalized virus (**Figure 3C**). Quantitation of IAV NP staining revealed that ~40% of control cells but not those treated with SP-A accumulated viral NP in the nucleus by 4 hrs. after infection, whereas only 5% of SP-A treated cells showed any intracellular NP staining 4 hours after infection (**Figure 3D**). Of the NP positive cells in the SP-A treatment group, most NP staining exhibited a perinuclear pattern rather than nuclear localization (**Figure 3C**). These results indicate that SP-A interferes with the kinetics of IAV uptake, transport, and HA function in the endosomal compartment.

**Figure 4:**
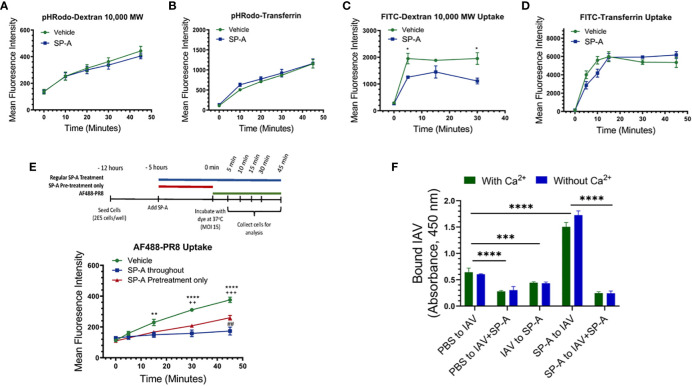
Differential reduction in uptake of endocytic cargo and binding of IAV PR8 to cell membrane in the presence of SP-A. Endocytic and binding experiments were performed using RAW264.7 cells **(A-E)** or isolated RAW264.7 membrane **(F)**. Raw264.7 cells were incubated with Dextran 10,000 or transferrin conjugated with either the pH sensitive dye pHRodo **(A, B)** or FITC **(C, D)** in 1:1 DMEM in the presence or absence of SP-A, for 15 minutes at room temperature. The media were replaced with dye-free 1:1 DMEM and cells incubated with PHRodo- and FITC-labeled molecules were chased at 37°C for 10, 20, 30, and 45-minutes and 5, 10, 15, 30, and 45-minutes at 37°C to monitor rates of endosomal acidification and uptake, respectively. **(A, B)** SP-A does not alter acidification rate for either Dextran **(A)** or transferrin **(B)** tracked endosomes, N=6. **(C)** SP-A reduced level of FITC-Dextran over the first 5-minutes, whereas SP-A appeared to delay uptake of FITC-transferrin between 5-20-minutes reaching a similar plateau after 30-minutes. Differences were significant for FITC-Dextran only. N=6, *p<0.05. **(D)** and **(E)** SP-A suppresses uptake rate of Alexa Fluor488-labeled PR8. RAW264.7 cells were incubated with IAV with SP-A throughout infection or pre-treatment only. Cells were infected with Alexa Fluor488-labeled PR8 at MOI=15. The uptake of the labeled virus was monitored for 5, 15, 30, and 45-minutes at 37°C by flow cytometry. N=6 per condition per time point, **p < 0.005 and ****p < 0.001 for vehicle vs SP-A throughout; ++, p < 0.005 and +++, p<0.0005 for vehicle vs SP-A pretreatment; ^##^, p < 0.05 for SP-A throughout vs SP-A. **(F)** To assess differences in membrane binding, immulon-2 flat bottom plates were coated overnight at 4°C with 12.5 μg/ml of Raw264.7 cell membranes and then washed and incubated with PBS, 5 x 10^6^ ffc of PR8, or 50 μg/ml SP-A for 2-hours at room temperature. The membranes were then washed, and incubated for an additional 2-hours with PR8, pre-formed PR8+SP-A complex, or SP-A to evaluate PR8 binding in a combination of conditions shown on the x-axis on panel F graph. Plates were washed and bound PR8 visualized spectrophotometrically using a polyclonal anti-HA antibody and HRP-conjugated anti-rabbit antibody as described in Materials and Methods. N=8, ****p < 0.00001, ***p < 0.001. Statistical differences were assessed by 2-way ANOVA.

To investigate whether SP-A alters acidification of endocytic vesicles, the pH-dependent dye pHRodo conjugated to various endocytic cargo, specifically transferrin and dextran, was used to measure endosomal pH changes following receptor- and fluid phase-mediated uptake, respectively. Cells were incubated with the dye for 15 minutes at room temperature in the presence or absence of SP-A, at which point the dye-containing media was removed and replaced with dye free media. Dye uptake was chased at 37°C, harvested at 10, 20, 30, and 45 minutes of incubation and analyzed for fluorescence intensity of the dye. These experiments showed that SP-A treatment did not significantly alter the acidification of endocytic cargo for either transferrin or Dextran (**Figures 4A, B**). Labeling with the pH stable dye FITC, and incubating the dye with the cells at 37°C for the indicated time, however, showed that SP-A reduced the level of internalized FITC-Dextran (**Figure 4C**). Uptake of FITC transferrin was moderately delayed over the first 15 min with a similar plateau after 20 min, indicating that SP-A suppresses early endocytic events but not recycling of endocytic cargo (**Figure 4D**). Furthermore, the presence of SP-A prior to or both before and during infection significantly reduced the uptake rate of AF488 conjugated IAV PR8 (**Figure 4E**).

To determine whether SP-A interferes with binding to the cell-surface, we performed IAV binding assay combinations using isolated cell membranes in the presence or absence of calcium (**Figure 4F**). Preformed IAV-SP-A complex reduced binding by 60% compared to IAV alone, indicating that SP-A interferes with binding of IAV. Sequential addition of SP-A after IAV binding also reduced binding by 40%, suggesting that SP-A can displace bound IAV from sites with low binding affinity or mask epitopes of the anti-IAV antibody used to detect bound virus. The binding affinity for influenza HAs ranges from 1 mM to 1 μM (81–84) or 10 to 100-fold lower than the binding affinity of SP-A to HA (**Figure 2F**). Sequential addition of SP-A before IAV, however, enhanced IAV binding by more than 3-fold. This enhanced binding was blocked by pre-formed SP-A-IAV complexes, indicating direct binding to membrane bound SP-A. The increased binding of IAV to membrane bound SP-A, however, was not observed when live cells were pre-treated with SP-A (**Figure 4F**, **Figures 1A-C, E, F**, **Figure 3C**), indicating that SP-A inhibits IAV by affecting macrophage function rather than accumulating on the cell-surface of live cells.

Taken together, these results indicate that SP-A impedes uptake and transport of IAV to acidified vesicles delaying delivery of viral genomes into the cytosol of macrophages.

### 3.4 SP-A enhances susceptibility to IAV infection

**Figure 5:**
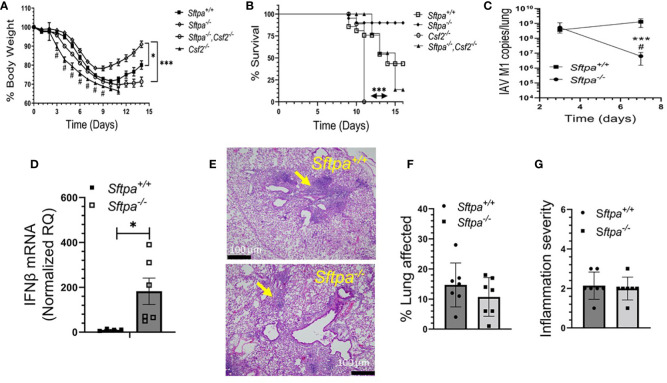
Disruption of SP-A improves recovery and clearance of IAV infection in mice. *Sftpa^+/+^* (n=14), *Sftpa^-/-^* (n=31), *Csf2^-/-^* (n=9) and *Sftpa^-/-^, Csf2^-/-^* (n=27) mice were infected with 1000 ffc (1 LD50) of IAV PR8 *via* the intranasal route. Body weight and survival were monitored daily. Total RNA was extracted from whole lung to measure viral RNA and IFNβ. **(A)** Lack of SP-A in *Sftpa^-/-^* mice enhances body weight recovery of *Sftpa^-/-^* mice compared to *Sftpa^+/+^, Csf2^-/-^* and *Sftpa^-/-^,Csf2^-/-^* mice (*p < 0.05, *Sftpa^-/-^* vs. *Sftpa^+/+^* days 9-14, ***p < 0.001 *Sftpa^-/-^* vs. *Csf2^-/-^* and *Sftpa^-/-^,Csf2^-/-^* mice), reduces body weight loss and mortality in the absence of GM-CSF (*p<0.05 *Sftpa^-/-^* vs. *Sftpa^+/+^* and ***p<0.001, Csf2^-/-^ vs. *Sftpa^-/-^, Csf2^-/-^*) **(A, B)**, enhances IAV clearance from the lung (***p < 0.001 *Sftpa^-/-^* vs. *Sftpa^+/+^* on day 7, ^#^ p < 0.05 Sftpa*^-/-^* day 7 vs. day 3 **(C)** and enhances expression of IFNβ as measured on day 3 after infection, n=6 mice, *p < 0.05 **(D)**. The histopathology **(E)** and histopathology scores **(F, G)** *Sftpa^-/-^* and *Sftpa^+/+^* consisting of perivascular cuffing (yellow arrows) and interstitial inflammation 14 days after infection were similar. Representative hematology and eosin stained tissue sections for *Sftpa^+/+^* (upper panel) and *Sftpa^-/-^* (lower panel) lungs in **(E)** were captured at 20x magnification. For **(F, G)** n=6 mice per group. Percent lung affected **(F)** and inflammation severity **(G)** scores shown are the mean observation from 5 microscopic fields from each lobe per mouse.

To evaluate whether SP-A alters the course of IAV infection *in vivo*, WT and SP-A-deficient mice were infected intranasally with a sub-lethal dose of IAV H1N1 PR8 and the severity of infection was monitored by comparing body weight and viral burden. Body weight recovery improved in SP-A-deficient (*Sftpa^-/-^*) mice compared to the WT (*Sftpa^+/+^*) controls (**Figure 5A**). Furthermore, lack of SP-A enhanced viral clearance between 3 and 7 days after infection compared to persistent infection in WT mice in this time period (**Figure 5B**). Lack of SP-A enhanced expression of anti-viral IFNβ 3 days after infection (**Figure 5C**). The lung histopathology of recovering WT and SP-A-deficient mice displayed perivascular cuffing and interstitial inflammation with lymphocytic aggregates, although smaller interstitial nodules were more prominent in the latter (**Figure 5E**). The overall histopathology scores, however, were similar (**Figures 5F, G**). Previous studies showed that GM-CSF inhibits SP-A and lipid binding (85, 86) and alters binding behavior of SP-A to alveolar macrophages (66, 86). Specifically, increase in GM-CSF levels inhibits high affinity SP-A binding whereas lack of GM-CSF results in low affinity binding, indicating that GM-CSF concentration alters the composition of low and high affinity binding sites for SP-A on alveolar macrophages. Furthermore, lack of GM-CSF results in lethal IAV infection, whereas application of high levels of GM-CSF improve survival (30–32). Therefore, we asked whether SP-A alters the lethal phenotype of GM-CSF-deficient mice. **Figures 5A, B** show that lack of SP-A in the GM-CSF (*Csf2^-/-^*)-deficient genetic background reduced body weight loss and improved prognosis for survival. These results indicate that SP-A suppresses viral clearance and enhances susceptibility to influenza infection in the presence of inadequate levels of GM-CSF *in vivo*.

## 4 Discussion

Previous studies reported that the pulmonary collectins, SP-A and SP-D, inhibit IAV infection through steric hindrance mechanisms that interfere with the ability of the virus to attach to sialic acid receptors on the membrane of host cells. By virtue of their multimeric nature, SP-A and SP-D binding can lead to agglutination facilitating uptake and clearance of the virus by macrophages. SP-D binds a mannose rich glycoconjugate proximal to the receptor binding site of HA that can be lost through antigenic drift or reassortment of the virus, resulting in IAV evading neutralization by SP-D. SP-A, on the other hand, offers its own sialylated glycoconjugate, which trap the virus but can be cleaved off by influenza neuraminidase resulting in the virus escaping clearance through SP-A-mediated agglutination and clearance (87).

Here, however, we report that SP-A plays a more complex role not only in the way it interacts with HA, but also by interfering with the endocytic process of virus uptake in macrophages. We found that the interaction of SP-A with HA is not strictly dependent on its glycoconjugate, and that binding to HA does not involve a carbohydrate-dependent mechanism as indicated by binding to de-glycosylated HA or the lack of an effect on binding in the presence of EDTA. Binding of de-glycosylated SP-A was similar to native SP-A at concentration above 10 μg/mL. The physiological concentration of SP-A sampled in BAL was estimated at 9.6 μg/mL in children and 3.6 μg/mL in adults (88). Accounting for a 100-fold dilution of surfactant in the BAL fluid, the local concentration of SP-A in the alveolar lining fluid would exceed 300 μg/mL (89, 90), a concentration that would favor a protein interaction mechanism. Furthermore, pre-incubation of SP-A with the virus before, but not during, the time of infection suppressed infection, suggesting that the liquid phase interaction at local SP-A concentration may interfere with HA and SP-A binding their receptor on macrophages. SP-A has been shown to inhibit infection of A549 alveolar type II-like epithelial cells pandemic H1N1 and H3N2 strains of influenza (58), suggesting that our findings may also apply to infection in alveolar type II epithelial cells. In this context, pH-dependent structural re-arrangement of HA influences virus infectivity, pathogenicity, and transmission (9, 10, 91). Here, SP-A delayed detection of the pH-dependent conformational epitope that is exposed in acidified endosomes. Prolonged exposure to acidic pH when the target membrane is not available for fusion leads to virus inactivation (9, 10, 91). Acidic conditions in the lung are encountered during endosomal trafficking, transport of HA during secretion in the Golgi (92), and in the extracellular fluid from respiratory acidosis that occurs during influenza-induced progression of acute respiratory distress syndrome (ARDS) (93). Future studies are, therefore, needed to define the binding parameters, interaction domains, and stability of the interaction under acidic conditions. Of note, human SP-A2 polymorphisms were linked to development of ARDS during the H1N1 swine influenza pandemic (57). Therefore, it is possible that the interaction of HA with SP-A may allow the virus to escape opsonization and inactivation under acidic conditions that may be encountered extracellularly during inflammation or during co-trafficking of the virus and SP-A in secretory pathways of alveolar type 2 epithelial cells (92). To this end, our finding that lack of SP-A enhances recovery in association with reduction in viral burden and, furthermore, that lack of SP-A reduces morbidity in the presence of GM-CSF (CSF2)-deficiency supports the idea that the interaction of IAV with SP-A sustains the IAV infection.

**Figure 6:**
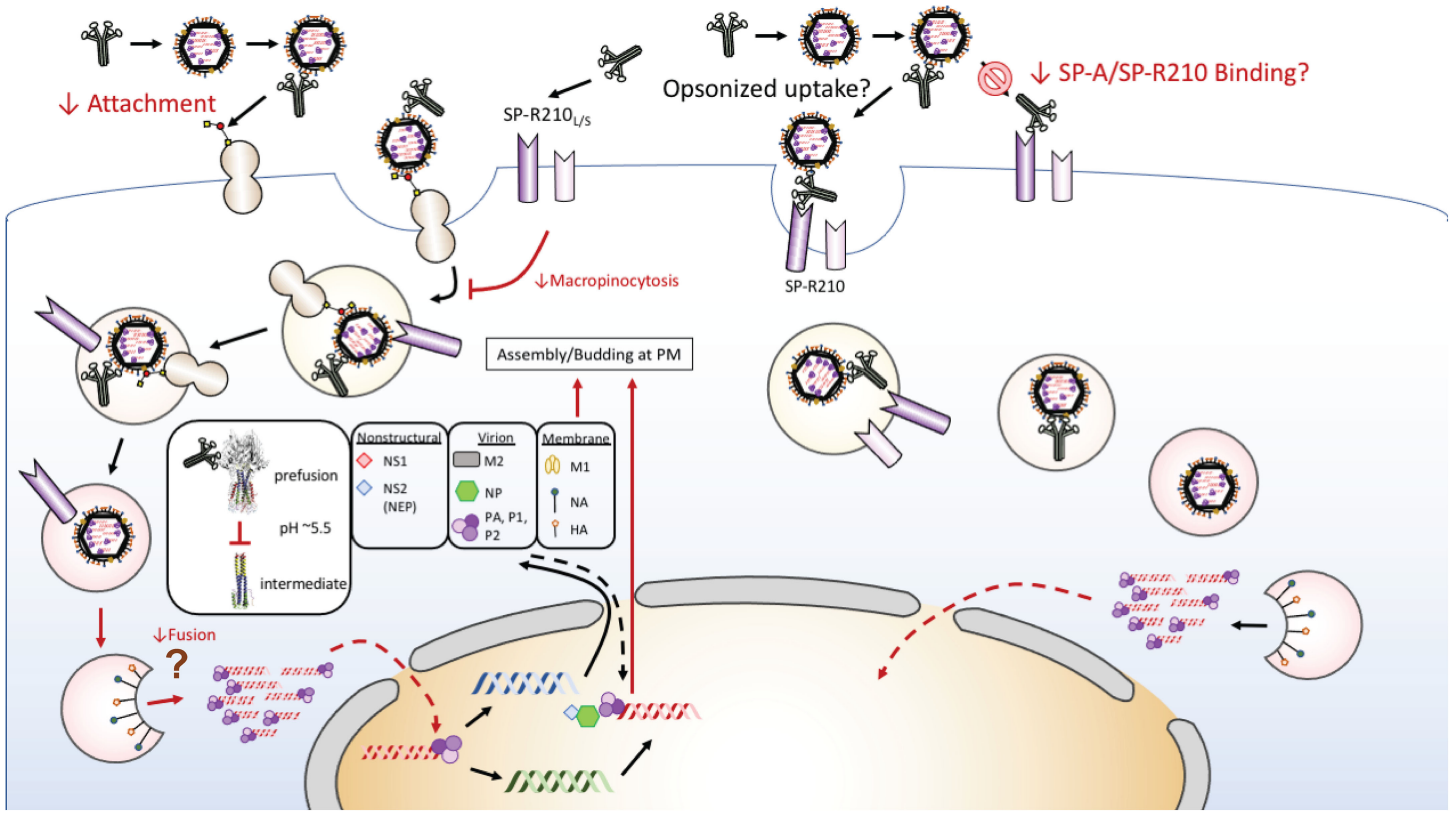
Proposed model of SP-A-IAV interaction in macrophages. Multi-site inhibition of IAV infection of macrophages occurs through indirect inhibition of macropinocytic entry, delayed sorting to acidified endosomes or steric hindrance of endosomal fusion of HA, and reciprocal inhibition of cognate receptor binding. A direct mechanism by the SP-A receptor SP-R210 may mediate endocytic entry and result in permissive infection that may be deployed by different strains of IAV along a gradient of SP-A levels and oligomeric forms towards the lower respiratory tract and over the course of infection.

Although ATII cells are the primary host for productive IAV in the distal lung *in vivo* (11), AMs are critical for the clearance and immune response to IAV infection (19, 22, 23, 30, 94–97). Furthermore, studies showed that SP-A modulates endocytic processes and signaling including endocytosis (77, 98–101), phagocytosis (69, 102, 103), and macropinocytosis (70) in macrophages through the SP-R210 receptor. SP-A itself is internalized *via* clathrin-mediated endocytosis leading to lysosomal degradation in macrophages (104–106). SP-A-mediated endocytosis enhances clathrin expression and endosomal suppression of NFκB activation through stabilization of the inhibitory subunit IκB and targeting of TLR4 to lysosomes for degradation through signaling mechanisms that involve activation of casein kinase 2 (CK2), protein kinase (PKC), and RAC(Rho family)-alpha serine/threonine-protein kinase (Akt1) (101), suppressing macrophage activation. The rate of SP-A uptake into lysosomes depends on activation of PKC and Akt1 (105). CK2 is also involved in SP-A-mediated phagocytosis (107). SP-A enhances phagocytosis through both direct opsonization and indirectly by enhancing expression and activation of a variety of non-opsonic and opsonic receptors such as scavenger and IgG receptors, respectively (68, 69, 102, 108). These activities could help reduce activation of antiviral mechanisms by macrophages. Here, the presence of SP-A prior to or both before and throughout infection, as occurs *in vivo*, however, suppressed the uptake rate of fluorescently labeled IAV. SP-A did not affect transport of endocytic cargo to acidified endo-lysosomes. SP-A, however, suppressed uptake level of Dextran 10000, which is internalized by both clathrin- dependent endocytosis and clathrin-independent macropinocytosis (109, 110), whereas internalization of transferrin, which is clathrin-dependent, was largely unaffected. Macrophages utilize macropinocytosis and endocytosis to differentially process and present antigen through MHC-I and MHC-II, respectively (111). Therefore, by modulating uptake and endosomal processing of the virus, SP-A may also shape the adaptive immune response to influenza by AMs. Additionally, immunofluorescence data suggests that SP-A may partially modulate access of IAV HA to endosomal membrane delaying release of the viral genome to the macrophage cytosol, while the uptake study of AF488-PR8 indicates that SP-A also blocks uptake of IAV, even if SP-A is not present concomitantly with the virus. This, alongside the study assessing how timing of SP-A addition affects IAV infection and binding in live cells and isolated cell membranes, indicates that SP-A can interact with the macrophages to alter IAV uptake as well as by interacting with IAV to reduce IAV infection of macrophages. Taken together, the data presented here show that SP-A attenuates IAV infection in macrophages in a step upstream of viral genome release (**Figure 6**).

Additional investigation is needed to reconcile discrepancies between the present study and among the studies reported by Levine et al. (63) and Hawgood and colleagues (62, 112). We report that SP-A suppresses clearance of H1N1 strain PR8. Levine et al, however, reported that lack of SP-A results in increased viral burden from infection with the H3N2 Phil82 strain (63). This is unexpected given the vulnerability of this strain to SP-D-mediated neutralization. A Th1-biased immune response shown in their study, however, suggests that SP-A shaped the humoral immune response, although the level of neutralizing antibodies was not evaluated. A high inoculum dose was needed, however, to induce infection with the Phil82 strain in mice. In contrast, Hawgood and colleagues reported that clearance of the mouse-adapted strain X-79, a re-assorted PR8 strain carrying the H3N2 Phil82 HA (113) was similarly impaired in both SP-D-deficient and SP-A-SP-D double-deficient mice, but not in SP-A-deficient mice (62, 112), consistent with vulnerability of this strain to SP-D neutralization. Lack of SP-A, however, blunted the Th1 immune response to SP-D-resistant X-79Δ167 mutant, as indicated by suppression of IFNγ with a marginal reduction in survival with increasing inoculum dose, and no difference in viral burden (112) when compared to the induction of IFNγ after infection with the H3N2 Phil82 strain shown by Levine et al. (63). Even though the X-79 HA is derived from the H3N2 Phil82 strain, SP-A was shown to neutralize hemagglutination by H3N2 Phil82 (114) but not by X-79 (62), suggesting that X-79 lost the ability to bind SP-A (62), although it was not indicated whether this occurred during reassortment or adaptation through passage of X-79 in the mouse host. A dual evasion of SP-A and SP-D binding would favor X-79 infection of macrophages through cell-surface lectin receptors (47–51). The present findings show that, in the absence of SP-A, H3N2 Phil82 reached full infection of the macrophage cell culture monolayer between 6-12-hours compared to 12-24-hours for H1N1 PR8. Therefore, comparative studies are needed to discern the role of SP-A on endocytic entry mechanism and host responses to infection with H1N1 and H3N2 IAV. In this regard, we also show that lack of SP-A enhanced early expression of anti-viral IFNβ, suggesting enhanced innate recognition of the virus. In this regard, it is possible that SP-A alters endosomal sorting that interferes with recognition of the virus by endosomal toll-like receptors resulting in a suboptimal anti-viral response or diverting the virus to lysosomes for degradation reducing the number of viral genomes that escape to the cytosol. Our previous studies showed that SP-A is a paracrine effector of the anti-inflammatory isoform of the SP-A receptor SP-R210_L_ (70); disruption of the SP-R210_L_ isoform in macrophages enhances endosomal recognition and activation of antiviral response to IAV through activation of IRF7 (115). Therefore, comparative studies are needed to discern the role of SP-A on endocytic entry mechanism and host responses to infection with H1N1 and H3N2 IAV in macrophages with differential expression of SP-R210 isoforms that may be encountered during infection *in vivo*.

## 5 Conclusions

We demonstrate that SP-A presents as a dual extracellular and endosomal block that delays IAV infection and prolongs transit of the virus through the macrophage endosomal compartment *via* interaction with both macrophages and IAV hemagglutinin (**Figure 6**). Whether this interaction interferes with fusion of viral and endosomal membranes, is a matter of future investigation. Two additional SP-A effects that need to be addressed in future studies are whether prior exposure of macrophages to SP-A alters IAV binding to cell-surface receptors including competition with the SP-A receptor or whether the incubation of SP-A with the virus alters its viability. The interaction of SP-A with HA does not involve sugar binding as the major mechanism for either protein for the H1N1 PR8 strain. This deviates from previous findings that attributed the anti-influenza effects of SP-A to its ability to act as a sialic acid bearing viral sponge for IAV or as an opsonin, facilitating aggregation and clearance of the virus by macrophages (59–61), although each of these three mechanisms may be deployed to different influenza strains. Whether the SP-A-HA interaction results in reciprocal inhibition of each strain binding to their respective receptors on macrophages reducing receptor-mediated uptake remains to be determined. Our SPR experiments show a binding affinity of 100 nM for SP-A binding to HA. This would prevent overwhelming infection, as defined by reduced internalization of the virus, but also delay immune recognition. On the other hand, formation of non-neutralizing SP-A-IAV, that is, non-aggregated complexes may favor clathrin-mediated endocytosis *via* the SP-R210 receptor through which trafficking of a stable SP-A-IAV complex interferes with fusion of HA with the endosomal membrane delaying replication of the virus or by inhibiting non-specific internalization through macropinocytosis. In this regard, SP-A exists as a deca-octamer of six trimeric units as well as lower order oligomers that bind surfactant phospholipid head groups on curved surfaces of surfactant phospholipid membranes. This serves to support formation and maintenance of large aggregate surfactant lipids in the form of tubular myelin. The estimated distribution of lipid-bound and aqueous forms of SP-A is 9:1 (116). Therefore, IAV may encounter multiple oligomeric forms of SP-A in the alveolar lining fluid. It is not yet known whether pH-dependent conformational changes in SP-A affects dynamic structural rearrangement in HA during endocytic trafficking delaying viral replication. In this manner, SP-A may also protect the virus from inactivation during inflammatory acidification of the airway surface fluid. Furthermore, SP-A suppressed the magnitude of constitutive macropinocytic entry as indicated by reduction in uptake of FITC-Dextran. Macropinocytosis may operate to balance ingestion and degradation of tubular myelin but also alter endocytic sorting of the virus. Studied *ex vivo*, alveolar macrophages internalize and degrade SP-A rapidly (104, 105, 117). In the lung, SP-A associates with alveolar macrophages constitutively (118, 119). In this microenvironment, GM-CSF provides sufficient signal to catabolize surfactant avoiding excess accumulation of surfactant lipoprotein (65, 85). We found that pre-incubation of isolated membranes with SP-A increases binding of IAV and the deletion of SP-A in the absence of GM-CSF improve survival. In this context, GM-CSF provides optimal signals for endo-lysosomal sorting that is important for the degradation of both lipids and viral infection (120). To this end, the interaction of SP-A and GM-CSF receptor pathways (86) at the interface of homeostasis, viral infection and host defense and the role of SP-A-IAV interaction on the antiviral and inflammatory response warrants future investigation.

## Data availability statement

The raw data supporting the conclusions of this article will be made available by the authors, without undue reservation.

## Ethics statement

The animal study was reviewed and approved by Pennsylvania State University College of Medicine Institutional Animal Care and Use Committee. Written informed consent was obtained from the owners for the participation of their animals in this study.

## Author contributions

EY performed experiments, contributed to methodological and experimental design, graphed and analyzed data, and wrote original manuscript draft. LY and YC performed experiments, contributed to methodological and experimental design, and graphed and analyzed data. TU contributed to project management and *in vivo* experiments. HA evaluated and performed blind scoring of tissue histopathology. JY generated and mapped monoclonal antibodies to hemagglutinin. CG and ZK performed and analyzed surface plasmon resonance experiments. EH developed mouse lines. ZC led the study and contributed to conceptualization, formal analysis, funding acquisition, methodology, project administration, writing-review & editing. All authors contributed to the article and approved the submitted version.
