# Supplementary figures and images for: Surfactant protein A alters endosomal trafficking of influenza A virus in macrophages
Source: Front Immunol. 2023 Mar 7;14:919800. doi: 10.3389/fimmu.2023.919800 (PMC10028185; doi:10.3389/fimmu.2023.919800)

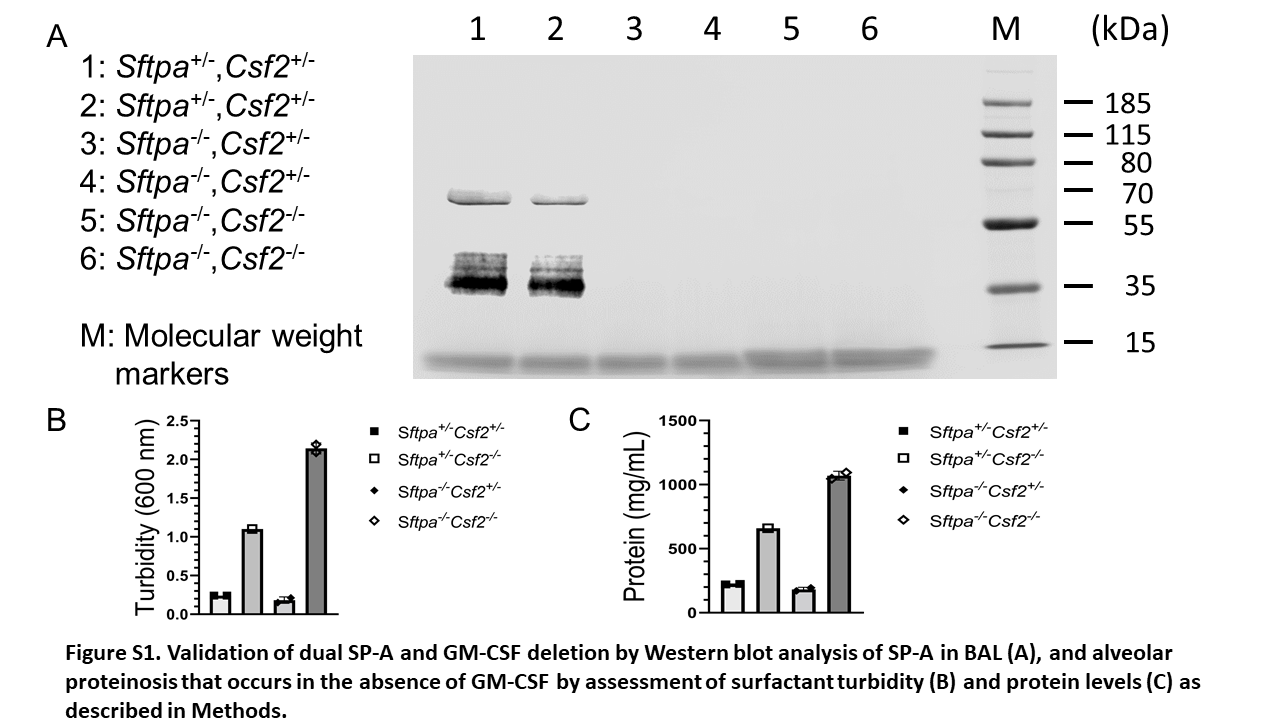

Supplement: Supplementary file 1 [file Image_1.tif]

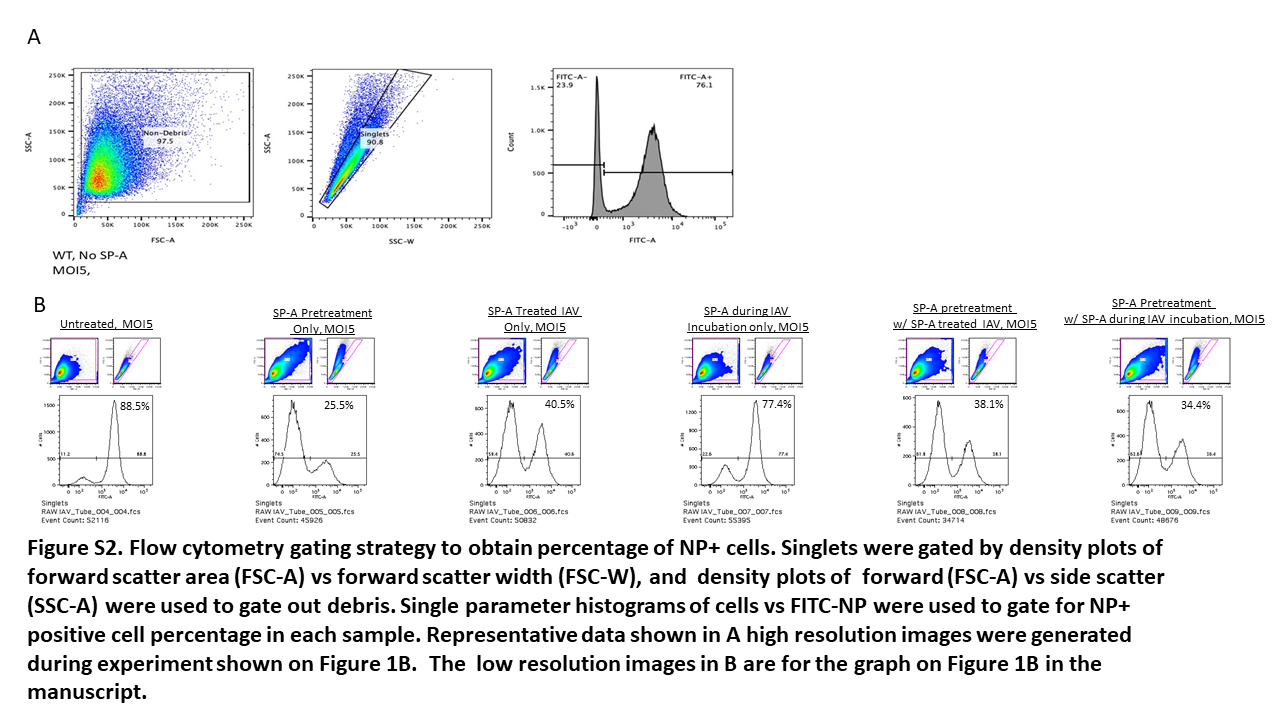

Supplement: Supplementary file 2 [file Image_2.tif]

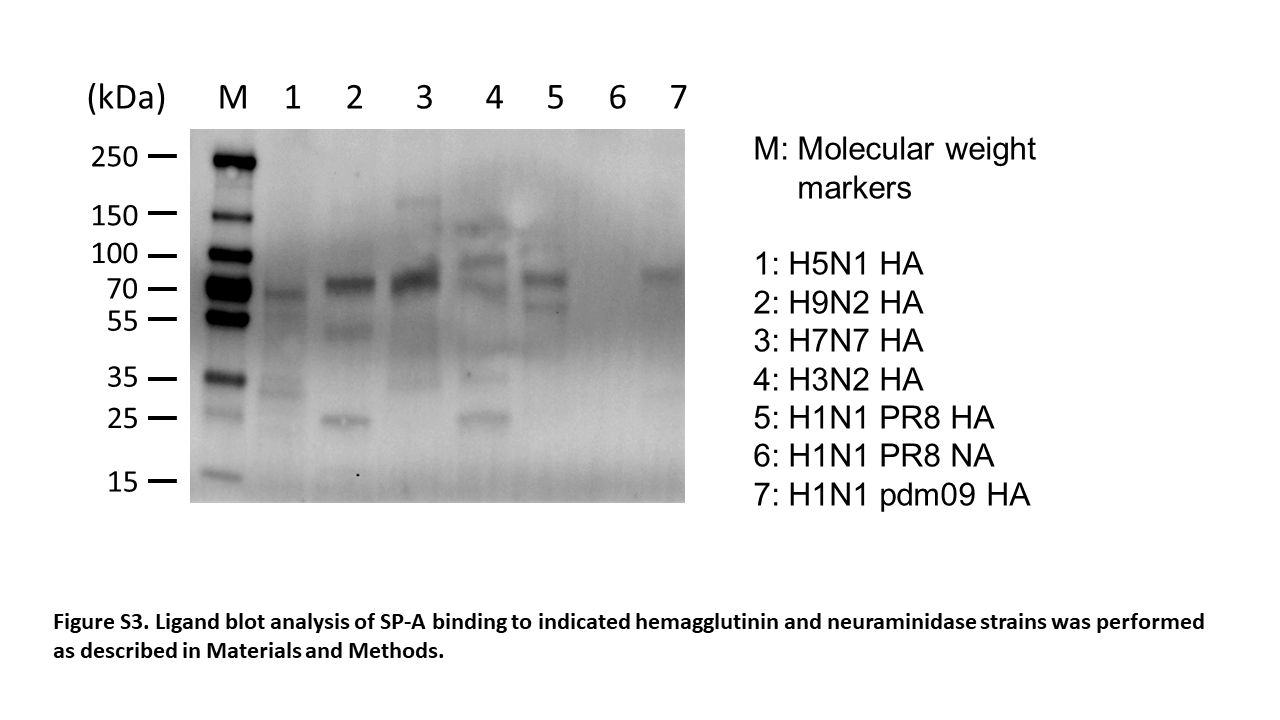

Supplement: Supplementary file 3 [file Image_3.tif]

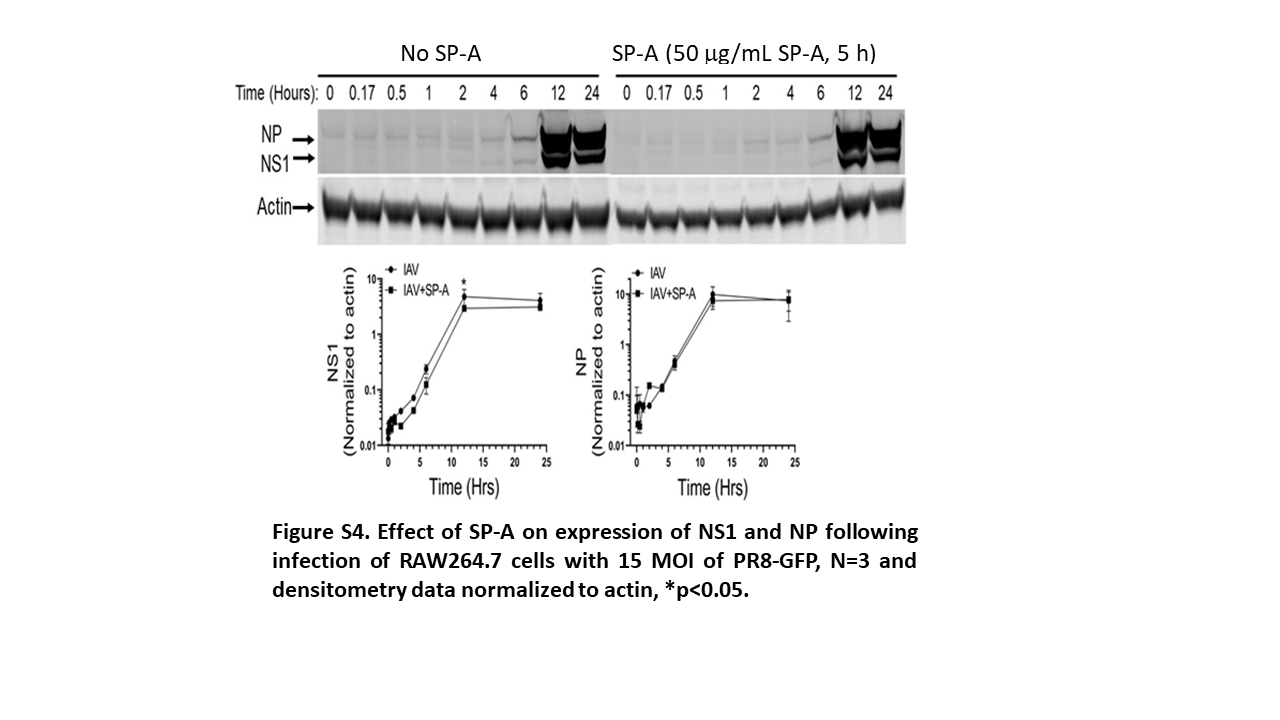

Supplement: Supplementary file 4 [file Image_4.tif]
